# Seasonality and Objective Physical Activity and Sedentary Behaviour among Older Adults from Four European Countries

**DOI:** 10.3390/healthcare11172395

**Published:** 2023-08-25

**Authors:** João Martins, Houshmand Masoumi, Vânia Loureiro, Margarida Gomes, Fortunata Ratinho, Tiago Ribeiro, Melika Mehriar, Marija Rakovac, Davor Šentija, Andrzej Bahr, Marta Tomczyk, Wojciech Dynowski, Roberto Solinas, Maria Grazia Pirina, Donatella Coradduzza, Giannangelo Boccuzzi, Birol Çağan, Ahmet Dalcı, Athanasios Papageorgiou, Soultana Smaga, Georgios Parisopoulos, Georgios Patsakas, Ioannis Meimaridis, Nuno Loureiro, Adilson Marques

**Affiliations:** 1 Faculty of Human Kinetics, University of Lisbon, 1649-004 Lisboa, Portugal; tiagodelgadoribeiro@gmail.com; 2 Center for Technology and Society, Technische Universität Berlin Germany, Kaiserin-Augusta-Alle, 104, 10623 Berlin, Germany; masoumi@ztg.tu-berlin.de (H.M.); mehriar@ztg.tu-berlin.de (M.M.); 3 Department of Transport and Supply Chain Management, College of Business and Economics, Kingsway Campus, University of Johannesburg, Johannesburg 2006, South Africa; 4 Instituto Politécnico de Beja, Rua Pedro Soares, 7800-295 Beja, Portugal; vloureiro@ipbeja.pt (V.L.); margarida.gomes@ipbeja.pt (M.G.); fortunataratinhogdl@hotmail.com (F.R.); nloureiro@ipbeja.pt (N.L.); 5 Faculty of Kinesiology, University of Zagreb, Horvaćanski Zavoj 15, 10000 Zagreb, Croatia; marija.rakovac@kif.unizg.hr (M.R.); davor.sentija@kif.unizg.hr (D.Š.); 6 Sports and Recreation Centre, Cracow University of Technology, Ul. Kamienna 17, 30-001 Kraków, Poland; andrzej.bahr@pk.edu.pl (A.B.); wojciech.dynowski@pk.edu.pl (W.D.); 7 Mine Vaganti N.G.O., Via del Vicolo del Fiore Bianco, 13/A, 07100 Sassari, Italy; president@minevaganti.org (R.S.); mvngo.board@gmail.com (M.G.P.); boccuzzi.giannangelo@gmail.com (G.B.); 8 Department of Biomedical Sciences, University of Sassari, Viale San Pietro 43/B, 07100 Sassari, Italy; donatella.coradduzza0@gmail.com; 9 Spor Elcileri Dernegi (SPELL), Yakinca Mh. Kenan Işık Cad. No: 14 Yeşilyurt, 44915 Malatya, Türkiye; birolcagan@hotmail.com; 10 Middle School Üzümlü, İnönü Univierstiy Hayriye Basdemir, İnönü Ünv. Merkez, 44000 Malatya, Türkiye; dalciahmet@gmail.com; 11 Northern Greece Physical Education Teachers’ Association (EGVE), Proxenou Koromila 51, 546 22 Thessaloniki, Greece; apapageor1@gmail.com (A.P.); soultanela@yahoo.gr (S.S.); gipariso@outlook.com (G.P.); geopat67@gmail.com (G.P.); ihmeima@gmail.com (I.M.); 12 CIPER, Faculdade de Motricidade Humana, Universidade de Lisboa, 1649-004 Lisboa, Portugal; amarques@fmh.ulisboa.pt; 13 ISAMB, Faculdade de Medicina, Universidade de Lisboa, 1649-004 Lisboa, Portugal

**Keywords:** seasonality, physical activity, sedentary behaviour, health, elderly

## Abstract

Objective: The present study aimed to explore the relationship between objective physical activity and sedentary behaviour with seasonality among a sample of older adults living in four European countries. Methods: A sample of 169 older adults living in Croatia, Greece, Portugal, and Poland (mean age = 72.2 ± 6.0, 68% female) had valid objective physical activity and sedentary behaviour data collected in different seasons of the year: spring and autumn/winter. Physical activity and sedentary behaviour were collected with accelerometers (ActiGraph, GT3X), over 7 consecutive days, in both periods. A valid record was defined as at least two weekdays and one weekend day with 10 hours of wearing time. Analyses were performed with IBM SPSS 28.0, using *t*-test, ANOVA, and binary logistic regressions. Results: Most older adults from the four countries met the physical activity guidelines in spring and autumn/winter. No significant variations were found across seasons for sedentary behaviour and physical activity both for light and vigorous intensity, regardless of sex, country, education, and body mass index (BMI). A decline in moderate physical activity intensity from spring to autumn/winter was found for those with lower education and higher BMI. Conclusion: The promotion of physical activity must be considered in programs to promote healthy aging throughout the year, especially considering the moderate intensity and those populations with higher BMI and lower educational levels.

## 1. Introduction

Physical activity plays a crucial role in maintaining overall health, well-being, and healthy aging [1]. Physical activity can be defined as any bodily movement produced by skeletal muscles that requires energy expenditure [2]. Its regular practice improves cardiovascular health and helps reduce the risk of heart disease [3]. Additionally, among other benefits, physical activity positively impacts mental health by reducing symptoms of depression and anxiety [4,5]. Physical activity also aids in weight management and the prevention of obesity [6]. Furthermore, active older people tend to benefit in terms of coordination, prevention of falls, reducing isolation, and maintaining social links [7,8]. On the other hand, physical inactivity and sedentary behaviour are associated with an increased risk of chronic diseases such as obesity, type 2 diabetes, cancer, and musculoskeletal disorders [9]. Understanding the risks of physical inactivity highlights the importance of incorporating physical activity into daily routines.

Physical activity recommendations for older adults include engaging in at least 150 min of moderate-intensity aerobic activity or 75 min of vigorous-intensity aerobic activity per week, or a combination of both, along with muscle-strengthening exercises at least two days per week [10]. Furthermore, older adults should carry out varied multicomponent physical activity focused on balance and strength training at moderate or greater intensity three or more days a week. However, adherence to these guidelines remains a challenge. Around a third of the world’s population is estimated to be non-compliant with global physical activity recommendations, with levels of physical inactivity being higher in high-income countries and among older adults [11].

Seasonality has emerged as a potential factor influencing physical activity levels. Seasonality refers to the cyclical changes in weather, daylight hours, and environmental conditions throughout the year [12]. These changes can significantly influence people’s behaviours and activities, including their engagement in physical activity [13].

Several factors such as temperature, precipitation, and daylight contribute to the seasonal variations in physical activity levels. Temperature is a critical factor affecting physical activity participation during different seasons. In colder months, individuals may be less inclined to engage in outdoor activities due to discomfort or concerns about exposure to cold-weather-related risks. On the other hand, warmer temperatures during spring and summer can facilitate increased participation in outdoor activities, including walking, cycling, and sports [14]. Even in young people this association is found, since playgrounds are usually outdoors and are where they usually engage in physical activity at school [15,16].

Precipitation, such as rain or snow, can also impact physical activity engagement. Inclement weather conditions may limit opportunities for outdoor activities and lead to decreased motivation for physical activity [17]. Safety concerns related to slippery surfaces or reduced visibility during precipitation can deter individuals from participating in outdoor physical activities. 

Daylight availability also influences physical activity behaviours. During winter, shorter daylight hours can limit the time available for outdoor activities, particularly after work. The reduced exposure to natural light and the tendency to spend more time indoors can impact individuals’ mood and motivation for physical activity. Conversely, longer daylight hours in spring and summer provide more opportunities for outdoor activities and may positively influence physical activity participation [18].

Physical activity and seasonality seem to be closely interconnected, as the changing seasons can significantly impact individuals’ engagement in physical activity. In brief, a recent systematic review focused on adults and older adults, proved that in summer and spring, compared to winter, physical activity levels tend to be higher [13]. Still in this review, some studies compared seasons more favourable for physical activity, such as spring/summer vs. autumn/winter, finding statistically significant differences that favour the aforementioned season of the year. However, further studies where the data are disaggregated by sex and other sociodemographic characteristics are needed (e.g., body mass index, education level), especially in the elderly population. Importantly, this systematic review proves that a very limited number of studies have been carried out in two or more countries, ranging out of the dichotomy summer/winter. The present work addressed, therefore, some of the above-identified limitations.

The influence of seasonality on physical activity has important implications for public health promotion. Understanding the influence of seasonality on physical activity is crucial for developing effective strategies to promote year-round physical activity and mitigate the potential barriers posed by different seasons. Strategies targeting specific barriers associated with each season may help individuals maintain regular physical activity habits regardless of the weather or environmental conditions. Therefore, in the present study, we sought to explore the relationship between physical activity and seasonality by discussing the factors influencing seasonal variations in physical activity and sedentary behaviour among the elderly population in four countries and the implications for public health promotion.

## 2. Methods

### 2.1. Study Design and Participants

This study, which involved data collection in two periods (spring 2022 and autumn/winter 2022) among the same older adults, is part the “Interventions in the Elderly’s Mobility Modes for Promotion of their Physical Activity and Fitness” (FITOLD) project. This project was funded by the European Union [Grant Agreement No 622623-EPP-1-2020-1-DESPO-SCP] under the Erasmus+ Sport project involved academic and non-governmental partners from 7 seven countries, namely Germany (coordinator), Croatia, Greece, Italy, Portugal, Poland, and Turkey. The FITOLD project took place between January 2021 and June 2023 and the main purpose was to identify the factors associated with the physical activity and physical fitness of older adults (aged 60 years or more), living in countries where further studies involving this population are less common and, therefore, needed.

Data were collected in six out of those seven countries (German partners did not collect data). However, in the present study, only data from four countries (Portugal, Croatia, Poland, and Greece) are used since a minimum of 30 persons per country with valid accelerometery data in both periods (spring and autumn/winter) was predefined as a minimum for these specific analysis—i.e., a large sample in statistical terms [19].

In each country, older adults aged 60 years or more were randomly invited to participate in the study through the network of each partner (fitness clubs, senior universities, other community organisations working with the elderly, etc.). In addition to the age, the following selection criteria were considered: being healthy or having a controlled heath situation; being able to understand and maintain a conversation; and walking ability without using a gait aid. For exclusion criteria, we used the following: not having a stable health situation; having reduced physical or mental abilities that limit their participation; having auditive or visual limitations; having fallen one or more times during the last year; living in collective residences; and declining to participate. After approval from the ethics committee, the older adults were first approached. The conditions of the research were presented in a friendly and personal environment. Written consent from older people and approval based on a brief screening of health risks for exercise was required for participation in the study.

In brief, from the four mentioned countries, a total of 676 (64.5% female; mean age 72.1 ± 5.5) participants answered the questionnaire (Croatia = 209, Greece = 203, Portugal = 94, Poland = 170). Of those, based on their self-reported availability to provide data on two distinct moments separated by several months, 239 older adults were selected and used the accelerometer in moment one (spring 2022). Among these, 202 had valid accelerometer data. Finally, out of all that used the accelerometer in moment two (autumn/winter 2022), only 169 older adults had valid data for both periods and were, therefore, included in the present study.

### 2.2. Measures

#### 2.2.1. Physical Activity and Sedentary Behaviour Data Evaluation and Accelerometry Data Collection

The ActiGraph wGT3X-BT triaxial accelerometer was used to estimate the time spent on all physical activity intensities, including sedentary behaviour (ActiGraph, GT3X model, Fort Walton Beach, FL, USA). 

In this study, the GT3X was used on an elastic belt on the right side of the hip at the level of the iliac crest. The participants were advised to wear the belt with the activity monitor on the right side during all waking hours, excluding the time spent in the sauna, bath, shower, or in other water activities, under their clothes. Participants were informed to wear the device for seven consecutive days, including two weekend days. The participants were informed to record the timing of and reasons for every occasion that the device was removed in a registration form. The accelerometers were delivered personally, following a protocol defined for the whole research group. Experienced members from the University of Lisbon trained the researchers during a 3 h recorded session, and continuous support was provided during the data collection and analysis. Additionally, before the official data collection for the project, a pilot phase for using the accelerometer was established and implemented. Data were collected for 5–10 people. All data were analysed, and the project partners met to share their doubts and find solutions. This pilot phase was important since not all partners had worked before with these devices and methodologies for collecting data.

Regarding the data processing phase and checking for validity, for wear time validation, periods of at least 90 consecutive 0 counts were considered non-wear time. Each day with a wear time >10 h was considered a valid day [20]. To be included in the analysis, the participants had to present at least three valid days (with at least one weekend day). 

For the quality control and harmonization process, two different approaches were adopted. First a decentralized analysis was performed by each country on their data, using the methodology mentioned above for preliminary validation of the data. Then, centralized reprocessing occurred with the highest data resolution using the methodology mentioned above. The accelerometer data processing was performed by the University of Lisbon team while using the Actilife software (version 6) with specific standardized procedures. The epochs were set to 15 s during the download, and the biometric data were filled. Both the ‘.agd’ and ‘.gt3x’ files were stored with each participant’s code as their file name during the download. The cutoff values used to define the intensity of physical activity and, therefore, to quantify the mean time at each intensity (sedentary, light, moderate, or vigorous) were as follows: sedentary: <100 counts·min^−1^; light: 100–2019 counts·min^−1^; moderate: 2020–5998 counts·min^−1^ (corresponding to 3–5.9 METs); vigorous: ≥5999 counts·min^−1^ (corresponding to ≥6 METs) [20].

#### 2.2.2. Sociodemographic Variables

Country, sex (male/female), age (years), education, height (m), and weight (kg) were self-reported and collected as sociodemographic variables using the questions of the European Social Survey [21]. The education level of the participants was collected by using the question: “About how many years of education have you completed, whether full-time or part-time?” The adapted answer options provided were (1) up to 9 years, (2) 10–12 years, and (3) more than 12 years. The body mass index (BMI) was calculated using the formula weight [kg]/height² [m²]. Cutoff values were defined following the World Health Organization cutoffs: normal weight (18.5–24.9), overweight (25–29.9), and obesity (30.0 and above) [22].

#### 2.2.3. Seasonality

Data were collected at two different moments of the year. Moment 1 of data collection occurred between March and May of 2022 (i.e., spring). Moment 2 of data collection occurred several months later, between mid-October and December (i.e., autumn/winter) of 2022. Table 1 shows the weather by month in each country and climate classification.

### 2.3. Data Analysis

First, descriptive statistics were calculated for all variables under study (mean and percentages). For continuous variables, normality analysis was carried out using the Kolmogorov–Smirnov test and the homogeneity of variances using the Levene test. As this was a large sample, we invoked the central limit theorem in cases where some of the assumptions were violated. The relationship between physical activity intensity levels and time spent sedentary (at moment one and moment two) and gender, country, education level, and BMI was calculated using student *t*-tests and ANOVA. Where differences between the study groups were determined, a Tukey post hoc analysis was run to identify the groups with statistically significant differences. Next, as there were some differences between the time spent on physical activity and sedentary behaviour in the two moments of data collection due to seasonality, we proceeded to calculate the difference between the two moments. We created a new variable that was the result of this difference. We then used this variable to see if there were differences according to gender, country, level of education, and BMI, using student *t-*tests and ANOVA. For each of the moments, we intended to verify which sociodemographic characteristics most explained compliance with the recommendations for physical activity. For each moment, a binary logistic regression model was created. All analyses were performed using IBM SPSS 28.0, with a significance level of 0.05.

## 3. Results

The characteristics of the participants are shown in Table 2. Most of the participants were women (68%), retired (91%), overweight or obese (59.9%), and met the recommendations for physical activity both in spring (61.5%) and autumn/winter (50.9%).

Table 3 shows the relationship between sociodemographic characteristics and sedentary behaviour and different intensity levels of physical activity in spring and in autumn/winter. There were no differences between men and women in the time spent sedentary or the different intensity levels of physical activity at the two times of the year. 

As for countries, in spring and in autumn/winter, Poland was the country where participants spent significantly more time being sedentary compared to Greece and Portugal. Only in the spring differences were noted in the average time spent on light physical activity, with Portugal being the country with the highest value (230.9 min, 95% CI: 213.4, 248.5), and significantly differing from Poland and Greece.

Regarding education, participants with higher levels of education spent significantly more time sedentary both in spring (543.3, 95% CI: 528.0, 558.5) and in autumn/winter (583.1, 95% CI: 565.9, 600.3), with differences being found between the “≤9 years” and “>12 years” groups.

BMI was not correlated with time spent sedentary and on physical activity in spring. However, in autumn/winter, those who were normal weight practised significantly more moderate physical activity and moderate-to-vigorous physical activity compared to overweight and obese older adults.

Table 4 present the time variation in sedentary behaviour and physical activity in spring and autumn/winter. It was found that there are no differences in terms of sex and country. As for education, the less educated showed the greatest variation (decrease) in moderate physical activity and moderate-to-vigorous physical activity, differing significantly from those with more than 12 years of education. When comparing participants concerning BMI, the older adults with obesity showed the greatest change (decrease) in moderate physical activity and moderate-to-vigorous physical activity when compared to those older adults who had a normal weight.

Table 5 shows the sociodemographic factors that explain compliance with physical activity recommendations. In spring (OR = 0.92, 95% CI: 0.86, 0.97) and autumn/winter (OR = 0.91, 95% CI: 0.86, 0.97), age was negatively associated with compliance with physical activity recommendations. Similarly, also in spring (OR = 0.90, 95% CI: 0.82, 0.99) and autumn/winter (OR = 0.84, 95% CI: 0.75, 0.93), BMI was inversely associated with compliance with the physical activity recommendations

## 4. Discussion

The present study analysed the impact of seasonality on objective physical activity and sedentary behaviour and explored how the potential sociodemographic factors related to sex, education level, BMI, and country may affect this relationship. Overall, it was found that more than half of the older adults met the physical activity guidelines, with age and BMI being significantly and inversely related to this outcome. Sedentary behaviour, light PA, and vigorous PA did not vary from spring to autumn/winter, regardless of sex, age, BMI, or country. However, a significant decline between seasons was identified for moderate and moderate-to-vigorous physical activity, but only for those older adults with lower education levels and with obesity.

Physical activity is important for a healthy aging and has many physical, mental, and social benefits for older adults [1]. Epidemiological studies estimate that, worldwide, about 70% of adults and older adults meet the physical activity guidelines, and that physical activity is normally lower in older age groups and among women [23,24]. Most of these conclusions, however, tend to be made using self-reported data of national representative samples, which differs from the methodology used in the present study. Sun et al. [25] conducted a systematic review, where the physical activity in older people was measured either using self-reported or objective measurements. The authors concluded that the percentage of older adults meeting the physical activity guidelines ranged from 2.4% to 83.0%. In Portugal, a study that used a representative sample of the population and that measured the physical activity levels with accelerometers has shown that 35% of older adults (men = 46%, women = 29%) reached the PA recommendation of 30 min/day. The differences in the data collection methods (e.g., self-reported, objective, criteria used to for data processing), the population characteristics (e.g., age, health status), the country, and the moments of data collection make it difficult to perform direct comparisons across studies [26]. However, by using objective measures, we found that the older people involved in this study were classified mainly as physically active, regardless of their sex and country, which is a positive indicator.

Several studies have explored the correlations of physical activity among the population [24,27]. For the older adults, Sallis et al. [27] concluded that the evidence of positive associations of younger age and male sex with higher physical activity is mixed. In our study, no differences in physical activity at any intensity were found based on sex. Importantly, BMI was inversely related to moderate-intensity physical activity and moderate-to-vigorous physical activity, especially in autumn/winter. This means that individuals with worse body composition seem to be a group at risk for reducing their moderate intensity and moderate-to-vigorous physical activity during these moments of the year and, therefore, might be considered for interventions. Given the additional challenges posed by the autumn/winter (e.g. reduced day light, slippery surfaces, rain, snow), promoting indoor activities for this group might be needed and of value, since they are the ones who might benefit the most from the physical activity increase. Indeed, almost 60% of our older adult participants were overweight or obese, which is of concern since they tend to perform less physical activity [24,28], and face many other adverse health and social consequences.

At the country level, the participants from Poland presented the highest levels of sedentary behaviour, either in spring or in autumn/winter. The Polish sample of older adults spent almost 10 h per day of their waking time being sedentary. Compared to the other countries included in this study, Poland presented the most challenging weather conditions (cold, snow, daylight) and this might have played a role in the high levels of sedentary behaviour found. This finding is of concern because exposure to high amounts of sedentary behaviour significantly increases the risk of all-cause mortality, cardiovascular disease, type 2 diabetes, and other health conditions [29].

The older adults of other countries also presented concerning levels of sedentary behaviour, all above 8 hours per day. From a practical point of view, it is important to have programs to help older people to reduce the time they spend sedentary and increase their light-intensity physical activity by moving more, more often. The WHO [30] recommends that every movement counts and that it is important to limit the amount of time spent being sedentary, which should be emphasised and promoted among the population. Furthermore, it should be highlighted that replacing sedentary behaviour with light-intensity physical activity performed in daily routines (e.g., walking around, climbing stairs) can benefit their health, as well as activities performed at other intensities, based on individual capability [30]. In the present study, the older adults with a higher educational level spent more time being sedentary either in spring or autumn/winter. This finding suggests that among older people, special attention must be given to those with higher educational levels. We can speculate that this might be because this group have additional access to and make use of technologies, as well as literacy and digital literacy, and might do less house-related labour work.

Regarding the impact of seasonality on physical activity and sedentary behaviour, contrary to the main findings present in the literature [13], no variations have been identified between spring and autumn/winter regarding sedentary behaviour, light physical activity, and vigorous physical activity. Most studies that use accelerometers or other objective measures for similar outcomes tend to focus on the differences between spring/summer and winter. In these situations, the physical activity levels tend to be higher in spring/summer and lower in winter [13,31]. For example, seasonality impacted physical activity variation in studies conducted in Japan, UK, and USA [25]. A similar finding emerged in a study conducted in Poland [32], but in a population with spinal cord injury. Although this seems to be a trend, in the literature there are also several examples of studies conducted in European contexts where the physical activity and/or sedentary behaviour did not vary with seasonality [33,34]. Again, direct comparisons across studies are difficult to establish because of the characteristics of the seasonality, of the samples, and of the methods of data collection. Indeed, the reduction in vigorous physical activity was not expected since the values are residual in both periods.

In the present study, variations were only found for moderate-intensity physical activity and moderate-to-vigorous physical activity among the lower educational group and those older adults with higher BMI. Obese older adults can face more challenges to be and keep active across time (e.g., amplified perceived barriers—self-efficacy, body image, low physical fitness, low movement efficiency, biomechanical stress, tolerance to effort, isolation, etc.). In those situations, based on the WHO recommendations [30], p. 11, it is important to highlight that “when not able to meet the above recommendations, older adults with chronic conditions should aim to engage in physical activity according to their abilities” and may seek a healthcare professional to obtain advice on the frequency, volume, and intensity of a positive and meaningful physical activity experience appropriate to their need and ability. If we have addressed the potential explanations for those with higher BMI, we can speculate that the other group with a lower education level, during the spring, can often be outdoors doing more home or labour-related work. When different conditions due to seasonality are imposed, this group may be forced to reduce their moderate physical activity levels significantly. An education campaign targeting this group, within the whole population, about the diverse opportunities to be and keep active during winter, might be important to promote healthy aging.

The present work has some strengths and limitations. As strengths we highlight (1) the focus on older adults from four European countries that are not usually represented in the literature concerning this specific theme; (2) the use of a piloted and standard methodology to collect objective physical activity and sedentary behaviour data, allowing valid and reliable assessments regarding duration, frequency, and intensity; (3) the innovative approach to explore the association of seasonality with diverse physical activity intensity levels and sedentary behaviour, in addition to physical activity prevalence; (4) the focus on less-often-studied seasons by focusing on spring and on the transition from autumn to winter. As for the limitations, we identify that (1) the samples were not representative, could be larger, and, therefore, the generalisation of these findings is limited; (2) the samples from Italy (which did not reach 30 participants with valid data in the two periods) and Turkey (data and partner’s colleagues were affected by the 2023 earthquakes) were removed; (3) a loss of participants between both periods was controlled, but occurred; and (4) the precise collection of objective seasonality indicators in the weeks that the data collection occurred in each country did not occurred and could have helped to strengthen the discussion of the findings.

## 5. Conclusions

Most older adults from the four countries involved in this study met the physical activity guidelines to benefit their health either in spring or autumn/winter. Older people with higher age and obesity should be targeted in interventions to increase physical activity. No variations were found across seasons for sedentary behaviour, or light and vigorous intensities of physical activity. The prevention of a decline in moderate physical activity from spring to autumn/winter must be considered in programs to promote healthy aging, especially among those older adults with higher BMI and lower educational levels.

## Figures and Tables

**Table 1:** Weather by month in each country and climate classification (1991–2021).

Country	Season	Month	Average Temperature (°C)	Rainy Days	Classification
Portugal (Lisbon) *	Spring	March	13.5	6	Hot summer Mediterranean climate
		April	15.2	7	
	Autumn/Winter	October	18.7	7	
		November	14.6	8	
		December	12.3	7	
Croatia (Zagreb)	Spring	March	6.6	9	Warm humid continental climate
		April	11.8	11	
	Autumn/Winter	October	12	9	
		November	6.9	10	
		December	1.8	10	
Poland (Krakow)	Spring	March	3.5	12	Warm humid continental climate
		April	9.3	10	
	Autumn/Winter	October	9.7	9	
		November	5.1	10	
		December	0.9	11	
Greece (Theassaloniki)	Spring	March	9.7	7	Cold semi-arid climates
		April	14.1	7	
	Autumn/Winter	October	16	5	
		November	10.9	6	
		December	5.5	6	

Note: * Nearest city from Grândola with available data; source: https://en.climate-data.org, consulted on 14 June 2023.

**Table 2:** Sociodemographic and physical activity characteristics of the participants.

Variables	% OR M ± SD
Age	72.2 ± 6.0
Sex	
Male	32.0
Female	68.0
Country	
Croatia	24.9
Greece	21.3
Portugal	34.9
Poland	18.9
Education	
Up to 9 years	24.9
10–12 years	27.2
More than 12 years	47.9
Occupation	
Employed	4.5
Retired	91.0
Doing housework	1.3
Other	2.6
BMI (kg/m^2^)	26.7 ± 3.8
BMI categories	
Normal weight	40.1
Overweight	44.3
Obese	15.6
Physical activity recommendations	
Achieve_1 (spring)	61.5
Achieve_2 (autumn/winter)	50.9

Note: %, percentage; M, mean; SD, standard deviation.

**Table 3:** Relationship between older adults’ sociodemographic characteristics and sedentary behaviour and different intensity levels of physical activity in spring and in autumn/winter (minutes/day).

Variables	Sex			Country				
	Male	Female	*p*-Value	Croatia	Greece	Portugal	Poland	*p*-Value
Sedentary beahviour_1 (m/day)	517.4 (495.6, 539.2)	525.0 (511.3, 538.7)	0.546	531.0 (509.1, 552.8)	507.7 (478.2, 537.1)	501.4 (482.4, 520.4)	567.5 (548.3, 586.8)	**<0.001 ^A^**
Light PA_1 (m/day)	204.9 (192.1, 217.8)	214.0 (202.5, 225.4)	0.344	215.0 (198.0, 231.9)	185.5 (170.6, 200.3)	230.9 (213.4, 248.5)	198.2 (183.2, 213.2)	**<0.001 ^B^**
Moderate PA_1 (m/day)	37.0 (28.3, 45.7)	31.4 (26.7, 36.1)	0.217	33.5 (26.9, 40.1)	33.5 (20.6, 46.3)	32.9 (25.6, 40.2)	32.9 (25.1, 40.7)	0.999
Vigorous PA_1 (m/day)	0.7 (0.0, 1.3)	0.3 (0.0, 0.6)	0.242	0.1 (0.0, 0.1)	1.0 (0.0, 2.1)	0.2 (0.0, 0.4)	0.6 (−0.4, 1.5)	0.090
Total MVPA_1 (m/day)	37.7 (28.6, 46.8)	31.7 (26.9, 36.5)	0.203	33.6 (27.0, 40.2)	34.5 (21.0, 48.0)	33.1 (25.8, 40.5)	33.4 (25.0, 41.9)	0.997
Sedentary behaviour_2 (m/day)	574.0 (551.8, 596.1)	558.5 (543.7, 573.2)	0.243	560.5 (537.5, 583.5)	566.0 (537.0, 594.9)	545.1 (525.2, 565.1)	598.1 (568.8, 627.4)	**0.026 ^C^**
Light PA_2 (m/day)	203.2 (189.4, 216.9)	215.5 (204.7, 226.3)	0.184	213.8 (199.1, 228.5)	200.6 (179.9, 221.2)	220.8 (204.1, 237.4)	204.1 (188.2, 220.0)	0.310
Moderate PA_2 (m/day)	31.5 (24.1, 39.0)	28.1 (24.1, 32.2)	0.385	30.1 (23.7, 36.5)	27.2 (19.9, 34.5)	28.3 (21.0, 35.6)	32.0 (24.0, 39.9)	0.843
Vigorous PA_2 (m/day)	0.2 (0.0, 0.3)	0.2 (0.0, 0.4)	0.924	0.1 (0.0, 0.1)	0.6 (0.0, 1.1)	0.1 (0.0, 0.3)	0.1 (0.0, 0.1)	**0.024 ^D^**
Total MVPA_2 (m/day)	31.7 (24.2, 39.2)	28.3 (24.2, 32.4)	0.390	30.2 (23.8, 36.6)	27.8 (20.2, 35.3)	28.4 (21.1, 35.7)	32.0 (24.1, 40.0)	0.875
**Variables**	**Education**				**BMI Categories**			
	**≤9 Years**	**10–12 Years**	**> 12 Years**	***p*-Value**	**Normal Weight**	**Overweight**	**Obese**	***p*-Value**
Sedentary beahviour_1 (m/day)	488.3 (463.7, 512.8)	517.5 (495.5, 539.4)	543.3 (528.0, 558.5)	<0.001 ^a^	526.5 (508.5, 544.5)	524.3 (506.8, 541.8)	511.6 (478.2, 545.1)	0.691
Light PA_1 (m/day)	227.4 (206.9, 247.9)	213.3 (194.5, 232.1)	201.3 (190.8, 211.9)	0.056	203.9 (191.9, 216.0)	217.0 (203.3, 230.8)	206.5 (178.3, 234.7)	0.380
Moderate PA_1 (m/day)	39.0 (26.8, 51.3)	28.7 (22.0, 35.5)	32.7 (27.8, 37.6)	0.212	35.9 (29.8, 42.1)	31.1 (25.6, 36.7)	31.2 (14.5, 47.9)	0.554
Vigorous PA_1(m/day)	0.6 (0.0, 1.2)	0.1 (0.0, 0.2)	0.5 (0.0, 1.0)	0.382	0.6 (0.0, 1.3)	0.1 (0.1, 0.2)	0.7 (−0.2, 1.7)	0.186
Total MVPA_1 (m/day)	39.6 (26.9, 52.3)	28.8 (22.1, 35.6)	33.2 (28.1, 38.3)	0.205	36.5 (30.1, 42.9)	31.2 (25.7, 36.8)	31.9 (14.4, 49.5)	0.527
Sedentary behaviour_2 (m/day)	536.7 (510.3, 563.1)	553.2 (531.5, 574.9)	583.1 (565.9, 600.3)	**0.005 ^b^**	559.5 (539.4, 579.6)	573.5 (554.5, 592.4)	547.9 (519.0, 576.7)	0.323
Light PA_2 (m/day)	216.4 (196.1, 236.8)	222.2 (206.2, 238.3)	203.0 (191.6, 214.4)	0.145	202.6 (189.6, 215.6)	213.2 (200.0, 226.4)	224.3 (200.8, 247.8)	0.214
Moderate PA_2 (m/day)	26.3 (17.5, 35.0)	32.3 (24.9, 39.6)	29.0 (24.4, 33.5)	0.494	37.1 (30.1, 44.1)	25.9 (21.7, 30.1)	16.2 (11.3, 21.2)	**<0.001 ^c^**
Vigorous PA_2 (m/day)	0.2 (0.0, 0.4)	0.2 (0.0, 0.5)	0.2 (0.0, 0.4)	0.875	0.2 (0.0, 0.4)	0.2 (0.0, 0.4)	0.1 (0.0, 0.2)	0.829
Total MVPA_2 (m/day)	26.4 (17.6, 35.2)	32.5 (25.1, 39.9)	29.2 (24.6, 33.8)	0.489	37.3 (30.2, 44.3)	26.1 (21.9, 30.4)	16.3 (11.4, 21.3)	**<0.001 ^d^**

Note: variables ending in “_1” are referent to spring time; variables ending in “_2” are referent to autumn/winter time; BMI, body mass index; MVPA moderate-to-vigorous physical activity; PA, physical activity; m/day, minutes per day. Tukey HSD post hoc significant differences: ^A^ Poland different from Portugal and Greece; ^B^ Portugal different from Greece and Poland; ^C^ Poland different from Portugal; ^D^ Croatia different from Greece; ^a^ ≤9 years different from >12 years; ^b^ ≤9 years different from >12 years; ^c^ normal weight different from overweight and obese; ^d^ normal weight different from overweight and obese.

**Table 4:** Variation in sedentary behaviour and physical activity between spring and autumn/winter, by older adults’ sociodemographic characteristics (minutes/day).

	Sex			Country				
	Male	Female	*p*-Value	Croatia	Greece	Portugal	Poland	*p*-Value
Δ Sedentary behaviour (m/day)	56.6 (35.5, 77.6)	33.5 (18.7, 48.2)	0.078	29.6 (12.5, 46.6)	58.3 (17.9, 98.7)	43.8 (26.3, 61.3)	30.6 (4.8, 56.4)	0.365
Δ Light PA (m/day)	−1.7 (−15.9, 12.4)	1.6 (−7.4, 10.5)	0.686	−1.2 (−15.4, 13.1)	15.1 (−8.9, 39.1)	−10.1 (−19.9, −0.4)	5.9 (−8.8, 20.6)	0.096
Δ Moderate PA (m/day)	−5.5 (−12.5, 1.5)	−3.3 (−7.9, 1.4)	0.596	−3.4 (−7.7, 0.9)	−6.2 (−21.9, 9.4)	−4.6 (−9.0, −0.3)	−0.9 (−7.0, 5.2)	0.850
Δ Vigorous PA (m/day)	−0.5 (−1.2, 0.2)	−0.1 (−0.4, 0.2)	0.264	0.0 (0.0, 0.1)	−0.5 (−1.7, 0.7)	−0.1 (−0.3, 0.1)	−0.5 (−1.4, 0.5)	0.565
Δ Total MVPA (m/day)	−6.0 (−13.3, 1.4)	−3.4 (−8.2, 1.5)	0.553	−3.4 (−7.7, 0.9)	−6.7 (−23.1, 9.7)	−4.7 (−9.1, −0.3)	−1.4 (−8.0, 5.2)	0.865
	**Education**			**BMI Categories**				
	**≤9 Years**	**10–12 Years**	**>12 Years**	***p*-Value**	**Normal Weight**	**Overweight**	**Obese**	***p*-Value**
Δ Sedentary behaviour (m/day)	48.4 (26.6, 70.3)	35.8 (12.0, 59.5)	39.8 (21.2, 58.5)	0.750	33.0 (14.2, 51.8)	49.2 (30.0, 68.3)	36.2 (4.3, 68.1)	0.468
Δ Light PA (m/day)	−11.0 (−23.0, 1.1)	8.9 (−7.5, 25.4)	1.7 (−9.4, 12.8)	0.162	−1.3 (−13.2, 10.6)	−3.8 (−14.6, 6.9)	17.8 (−6.0, 41.6)	0.149
Δ Moderate PA (m/day)	−12.8 (−24.3, −1.3)	3.6 (−2.8, 9.9)	−3.7 (−7.7, 0.3)	**0.010 ^a^**	1.2 (−3.8, 6.2)	−5.2 (−10.0, −0.4)	−15.0 (−31.8, 1.9)	**0.020 ^c^**
Δ Vigorous PA (m/day)	−0.4 (−1.0, 0.2)	0.2 (−0.2, 0.5)	−0.3 (−0.9, 0.2)	0.340	−0.4 (−1.1, 0.3)	0.1 (−0.1, 0.3)	−0.6 (−1.6, 0.3)	0.182
Δ Total MVPA (m/day)	−13.2 (−25.2, −1.2)	3.7 (−2.8, 10.2)	−4.0 (−8.2, 0.2)	**0.011 ^b^**	0.8 (−4.6, 6.1)	−5.1 (−10.0, −0.2)	−15.6 (−33.3, 2.1)	**0.026 ^d^**

Notes: PA, physical activity, MVPA moderate-to-vigorous physical activity; m/day, minutes per day; BMI, body mass index; MVPA moderate-to-vigorous physical activity; PA, physical activity; m/day, minutes per day. Tukey HSD post hoc significant differences: ^a^ ≤ 9 years different from >12 years; ^b^ ≤9 years different from >12 years; ^c^ normal weight different from obese; ^d^ normal weight different from obese.

**Table 5:** Sociodemographic factors associated with meeting the physical activity guidelines in spring and in autumn/winter.

	Spring (Moment 1) OR (95% CI)	*p*-Value	Autumn/Winter (Moment 2) OR (95% CI)	*p*-Value
Age	0.92 (0.86, 0.97)	**0.004**	0.91 (0.86, 0.97)	**0.003**
Sex				
Male	1.00 (ref.)		1.00 (ref.)	
Female	0.69 (0.32, 1.51)	0.357	0.77 (0.36, 1.65)	0.504
Country				
Croatia	1.00 (ref.)		1.00 (ref.)	
Greece	0.38 (0.13, 1.11)	0.078	0.43 (0.15, 1.23)	1.116
Portugal	0.53 (0.19, 1.50)	0.232	0.64 (0.23, 1.79)	0.390
Poland	0.54 (0.18, 1.66)	0.282	0.94 (0.32, 2.73)	0.911
Education				
≤9 years	1.00 (ref.)		1.00 (ref.)	
10–12 years	0.37 (0.13, 1.08)	0.069	1.36 (0.47, 3.94)	0.571
>12 years	0.81 (0.29, 2.30)	0.692	0.69 (0.24, 1.99)	0.498
BMI	0.90 (0.82, 0.99)	**0.037**	0.84 (0.75, 0.93)	**<0.001**

Note: BMI, body mass index. CI, confidence intervals.

## Data Availability

The datasets generated during and/or analysed during the current study are available from the corresponding author upon reasonable request.
